# The Correlation between Enterprise Internal Control Quality and Research and Development Investment Intensity

**DOI:** 10.1155/2022/1788142

**Published:** 2022-06-24

**Authors:** Sheng Hu, Huiling Yang

**Affiliations:** ^1^School of Economics, Shandong University of Technology, Zibo 255000, China; ^2^School of Management, Jiujiang University, Jiujiang 332005, China; ^3^School of Accounting, Jiangxi University of Finance and Economics, Nanchang 330013, China

## Abstract

Innovation is the driving force behind enterprise development. Improving the quality of the internal control of enterprises and increasing the intensity of R&D investment are important ways to enhance the level of scientific and technological innovation. Based on the data of Shanghai and Shenzhen A-share listed companies from 2010 to 2019, in this paper, we empirically examine the correlation between the quality of internal control and the intensity of R&D investment by using the level of corporate cash holdings as an intermediary variable. We find that high internal control quality can improve cash-holding level and that improving cash-holding level will increase R&D investment intensity. That is, cash-holding level has a mediating effect between internal control quality and R&D investment intensity, while internal control quality can have a direct or indirect positive effect on R&D investment. Accordingly, this study has important theoretical significance and practical value for enterprises that seek to promote R&D innovation and improve their internal control.

## 1. Introduction

In the context of China's rapid economic growth, the topic of mass innovation remains hot. Since the 19th National Congress of the Communist Party of China proposed that innovation is the primary driving force for development, an increasing number of social organizations, enterprises, and public institutions have responded positively to these instructions, constantly pursuing innovation and making contributions to national economic development. Innovation continually upends traditional institutions, models, technologies, and ways of thinking. Thus, the innovation ability of enterprises has a crucial and decisive impact on the sustainability of scientific and technological innovation and the economic growth of China as a whole. R&D investment is the key factor that affects the innovation ability of enterprises. Increasing R&D investment intensity can improve the innovation efficiency of enterprises [1, 2]. However, due to the high cost, high risk, and long cycle of R&D activities, most enterprises in China have been repeatedly frustrated in their process of innovation. Due to R&D's financing constraints [3], high agency costs, high capital shortage costs, high information disclosure costs, and suboptimal incomes, these enterprises' enthusiasm for innovative research and development can gradually weaken. As a result, the intensity of R&D investment is reduced, and innovation behavior lacks support and guarantees. Consequently, what factors affect R&D investment intensity is a hot topic in both theoretical and practical circles [4]. Internal control plays an irreplaceable role in reducing agency costs, restraining the irrational behavior of company management, improving the quality of accounting information disclosure, reducing enterprise risk, and improving enterprise operation efficiency [5]. The main factors that hinder enterprises in the process of innovation and R&D include high agency costs, high information disclosure costs, and low risk returns. In this regard, the quality of internal control has a certain impact on enterprise innovation decisions. Therefore, studying the effect of internal controls on enterprises' research and development has important theoretical significance and practical value.

In 2008 and 2010, China issued the *Basic Standards for Internal Control of Enterprises* and the *Application Guidelines for Internal Control of Enterprises-Research and Development*, which play a positive role in guiding enterprises to improve their level of internal control and innovative R&D and to reduce their R&D losses. Internal control quality is related to proven factors affecting R&D investment, such as internal governance issues, financing costs, and agency costs. This raises the question of whether improving internal control quality affects R&D investment intensity. Thus, many scholars have carried out relevant studies and analyses. Some scholars [6–8] argue that internal control can directly or indirectly affect the R&D investment of enterprises and have put forward the “promotion theory of internal control,” which indicates that enterprises can improve the efficiency of enterprise operation and management and promote the progress of R&D through the construction or improvement in internal control systems. Other scholars [9–11] have suggested that, due to a cautious attitude, management may avoid costly and risky innovation activities. Strengthening internal controls may inhibit the intensity of R&D investment. Accordingly, the academic circle has not given a clear and unified conclusion regarding whether improving internal control quality promotes or inhibits the increase in R&D investment intensity, which is a major research in need of an urgent solution. Therefore, our first research focus in this paper is a discussion of whether improving internal control quality will affect the increase in R&D investment intensity and how internal control quality affects R&D investment intensity.

Compared to other investment decisions, most external investors are less willing to risk financially supporting enterprise research and development projects. Moreover, as it takes a long time to carry out research and development activities, and the results take time, it is easy for investors to lose confidence and exhibit slight behavior, making research and development activities more vulnerable to capital restrictions. Thus, R&D activities rely more on internal cash as a source of funding. According to relevant research statistics, from 2002 to 2014, the average cash holdings of listed manufacturing companies in China accounted for 29.5% of their total assets [12], and, from 2010 to 2016, the average cash-holding level of China's Shanghai and Shenzhen A-share nonfinancial listed companies was 19.5% [13]. Overall, cash holdings are high. Normally, companies will set aside enough cash for follow-up research and development activities, but there are typically some enterprises that lack effective capital management and control, which appear to have inefficient allocations of capital and resources due to certain factors, such as self-interested management motives of management and investment distortion, entailing that some R&D investment decisions are very short-lived. Hence, to explore the relationship between corporate cash-holding level and R&D activity in this paper, we adopt existing studies to analyze the relationship among internal control quality, cash-holding level, and R&D investment intensity, thereby extending the relevant literature on the influencing factors and paths of R&D investment intensity.

Based on the data of Shanghai and Shenzhen A-share listed companies from 2010 to 2019, we empirically examine the correlation between the quality of internal control and the intensity of R&D investment from the perspective of corporate cash-holding level. Through empirical research, two questions are answered. First, does high-quality internal control play a role in promoting R&D investment intensity? Second, does internal control quality indirectly affect R&D investment intensity by influencing cash-holding level? And does cash-holding level play a mediating role in the relationship between internal control quality and R&D investment intensity? In this paper, cash-holding level is introduced as a mediator variable to explore its effect on the relationship between internal control quality and R&D investment intensity, enriching the existing research perspective and offering a certain theoretical significance for further research on the influencing factors of innovation and R&D activities.

## 2. Related Works

Only with a clear understanding of the role of internal control can we obtain a better understanding of the influence mechanism involved in this research. The importance of internal control can be understood in two ways. One is checks and balances. As a control system for the board of directors, executives, and employees, a series of systems and measures in the process of internal control make precise use of checks and balances due to the internal division of labor within an enterprise, which can effectively reduce conflicts of interest between different subjects, alleviate agency problems within the enterprise, and ensure the efficient implementation of various decisions. Internal control can limit the authority of actors to control risks and improve efficiency [14]. The second is motivation. One of the objectives of internal control is to promote the realization of an enterprise's development strategy. Internal control can help enterprises clarify their actual positioning, gradually formulate reasonable plans, find the correct direction of action, and establish an effective incentive system to maintain the enthusiasm of members during some decision-making activities with long periodicity and high risk, playing a strategic guiding role and encouraging enterprises to progress in a positive direction via numerous positive aspects.

### 2.1. Internal Control Quality and R&D Investment Intensity

The influence path by which high-quality internal control can increase R&D investment intensity can be analyzed and explained with the three following aspects: First, high-quality internal control can reduce the implementation risk of innovative R&D activities and ensure that R&D decisions are directed, assured, and efficient. The implementation of any decision requires guidance toward goals and the coordination and support of a series of organizational relations. As strategic processes with long duration and unpredictable risk, innovative R&D decisions need to be even more firmly guided by a firm goal and assisted by a well-structured organization to ensure that these decisions can be carried out continuously. Internal control aims to achieve long-term development strategic goals and encourage enterprises to continue to innovate [15]. While providing the target direction, internal control can reduce blind or inefficient projects and enhance the efficiency of translating an R&D project into a company performance benefit through the supervision of all R&D projects and their activities. In this way, executives' interest in R&D projects will be aroused, and strategic decisions made by executives will be more inclined toward innovation and R&D. Thus, innovation ability and core competitiveness will be enhanced, and enterprises will have stronger vitality, broader development space, and higher profits. From the perspective of goal guidance, Jensen et al. [16] suggest that effective internal control can not only enable enterprises to clarify the direction and goals of R&D activities but also provide institutional investors with a clear and powerful direction, offering them a sense of confidence in the future that will help promote the implementation of enterprise innovation decisions. From the perspective of optimizing the consequences of internal control, Hu, Wang, and Zhang [17] indicate that technology-oriented small- and medium-sized enterprises should further improve their internal control system, provide high-quality financial information, and reduce information asymmetry to reduce the risks of technological innovation and improve the power and ability of R&D. From the perspective of corporate performance, Cassiman and Veugelers [18] discuss how internal control plays a positive role in promoting technological innovation, which extends from the essential role of internal control, that is, improving the efficiency of a company's management to create high performance. With a guarantee of good performance, a company will accelerate the implementation of its innovation decisions. Therefore, the extant research results clearly support the important role of internal control in promoting R&D investment.

Second, high-quality internal control can reduce the financing costs that are unfavorable to R&D and increase the financial support for R&D activities. Beneish et al. [19] indicate that, to provide better financing for innovative projects, enterprises need to present real financial reports or investment bases to investors to win their support and virtually reduce the cost of equity capital. High-quality internal control ensures that the best and truest side of an enterprise can be presented to prevent invalid financing events. The research of Kim et al. [20] also suggests that financing costs are affected by internal control. Improving the quality of internal control can resolve a series of problems that lead to high financing costs for enterprises, for example, by strengthening regulatory transparency for external investors and standardizing the financing decision-making process of senior managers to better reduce financing costs and alleviate the problem of insufficient R&D investment intensity.

Third, high-quality internal control can reduce the negative impact of agency problems on R&D activities. Due to R&D activities' long periodicity and large risk fluctuations, shareholders and senior managers need to pay close attention to the progress and changes of R&D tasks and make timely adjustment measures; however, the real implementation power rests with senior managers acting as agents. Agents may engage in behaviors that are detrimental to R&D investment because of risk aversion, laziness, or comfort. The Chinese *Enterprise Internal Control Evaluation Guidelines* thus urge the boards of directors and shareholders of enterprises to regularly conduct a comprehensive evaluation of the effectiveness of internal control and issue public evaluation reports. The implementation of this system can ensure that the agency problem between an enterprise's management, board of directors, and shareholders is taken seriously. Amid regular evaluation, to avoid damaging the reputation of its enterprise, a board of directors has to strengthen the supervision of and incentives for management, give full consideration to the funds needed for R&D projects, eliminate all negative situations—such as inadequate implementation measures, insufficient risk attention and control, and lack of properly directed project funds due to agents' selfish behavior—alleviate agency problems between managers and shareholders, and promote the implementation of funds needed for innovative R&D activities. Accordingly, high-quality internal control can limit the agency costs, information asymmetry, and self-interested senior management behavior that reduce R&D investment intensity.

Based on the above research, Hypothesis 1 is proposed as follows:

  H_1_: Internal control quality is positively correlated with R&D investment intensity. The higher the quality of internal control is, the larger the intensity of R&D investment is.

### 2.2. Internal Control Quality and Cash-Holding Level

According to previous studies on corporate cash reserves, the cash-holding level of many listed companies is often higher than 20%. Moreover, this amount for listed companies is typically at least 6 million yuan, as valuable as a factory, indicating that the cash-holding level of listed companies in China is relatively high. Because the development of Chinese capital market is not perfect, the external financing cost for many listed companies is high. Due to transaction, prevention, speculation, and other motives, companies will choose to reserve more cash to cope with financing difficulties. However, a high proportion of cash holdings requires a good cash flow management system because high cash reserves are easily squandered by senior executives. Specifically, when an enterprise has defects in its cash flow management system, its management may make excessive investments, thus reducing the level of corporate cash holdings. Cash flow management is an important unit of internal control activities. High-quality internal control can have a significant impact on the effective management of cash flow. The execution of internal control must be strong enough for enterprises to maintain a reasonable cash-holding level. Moreover, when the quality of internal control is improved, under clear and reasonable instructions, a system or program will restrain the irrational behavior of management due to overconfidence and will effectively reduce agency cost, improving the quality of accounting information disclosure and alleviating financing difficulties to improve cash-holding level.

To test this theory, many scholars have conducted empirical research on it. Among them, Kothari et al. [21] show that improving internal control quality can effectively reduce agency cost and restrain the irrational behavior of management to promote cash-holding level; that is, high-quality internal control can stimulate management's enthusiasm and increase its dedication to improving their enterprise's cash-holding level. Jaggi et al. [22] indicate that once internal control is improved and optimized, the excessive investment of management has an obvious decreasing trend and thus the motivation for cash holdings becomes more reasonable. Hollis Ashbaugh-Skaife et al. [23] argue that defective internal control aggravates the financing constraints of enterprises, leading to a vicious increase in financing costs and a reduction in cash holdings. Hall and Lerner [24] also show that the difference between internal and external financing costs inevitably causes financing constraints and that information asymmetry, principal-agent, and other problems indirectly reduce corporate cash holdings. However, the efficient operation of internal control plays a key role in reducing asymmetric information and increasing agency transparency, which can restrain the adverse factors and limit the consequences of financing constraints.

Therefore, Hypothesis 2 is proposed as follows:

  H_2_ : High internal control quality can improve corporate cash-holding level.

### 2.3. Internal Control Quality, Cash-Holding Level, and R&D Investment Intensity

There are two viewpoints in the research on cash-holding level and R&D investment intensity. The first is the “efficient hypothesis,” which argues that a high cash-holding level can boost R&D investment. When enterprises decide to undertake R&D activities, they must take the relevant funding sources and high-risk situations into account. Due to the lack of an effective supervision system and investor protection mechanism in the Chinese market, external investors have difficulty understanding the progress and expected results of R&D. They are more cautious of such investment with high uncertainty and underestimate the real value of R&D activities, creating so-called valuation risk for corporate R&D financing. Of course, there is also a common risk of information asymmetry. External investors have a certain degree of lag when comprehending enterprises' R&D decisions, which affects the timely arrival of R&D investment funds and may make enterprises miss some good opportunities. The existence of these external risks makes it difficult for R&D investment to be supported by external debt funds. In contrast, internal cash is easier to obtain, while enterprises usually reserve part of their internal cash for future emergencies, forcing them to rely more on internal cash in R&D financing. A high cash-holding level can provide the strong and stable financial support required for R&D investment, playing a strategic role in enterprise innovation. Through empirical research, Schroth and Szalay [25] indicate that whether an enterprise makes a decision to increase R&D investment intensity will largely depend on the amount of cash reserved inside the enterprise. From the perspective of payment methods, Almeida's research [26] shows that, amid high financing constraints, enterprises will adopt more noncash payment methods to carry out M&A activities, leaving a large amount of cash to support the funding of R&D investment activities. From the perspective of R&D smoothing, Brown and Petersen [27] demonstrate that cash holdings not only promote R&D investment intensity but also positively affect the sustainability of R&D activities.

Second, the “invalid hypothesis” viewpoint suggests that self-interested management may engage in rent-seeking behavior due to the large investment scale, long cycle, slow return, and uncertain expected return of R&D activities. In addition, there are agency conflicts between management and shareholders, while management tends to seek its own profits instead of supporting R&D activities. The research of Gray [28] shows that state-owned enterprise management in China prefers to invest retained profits and cash in short-term private projects with large control over R&D projects that benefit long-term productivity development. Harford [29] argues that, due to the strong liquidity of cash, it is more likely to be misappropriated by management and controlling shareholders into their own income via noninvestment or inefficient investment, resulting in false supportive cash inflow. At present, the internal management system of enterprises is gradually being optimized and improved, and the decision-making activities of their management are gradually becoming more transparent. Provided various conflicts and contradictions are resolved, the cash held by an enterprise can be more rationally used for R&D investment to support its innovation strategy. Therefore, we favor the “efficient hypothesis,” which argues that a high cash-holding level promotes R&D investment. The above theoretical analysis and literature review reveals that internal control quality can promote an improvement in corporate cash-holding level and that a high cash-holding level can promote R&D investment. Thus, internal control quality can indirectly affect R&D investment intensity through cash-holding level.

Therefore, Hypothesis 3 is proposed as follows:

  H_3_: The higher the quality of internal control is, the higher the level of cash holding is, and the larger the R&D investment intensity is; that is, cash-holding level has a mediating effect.

## 3. Research Design

### 3.1. Sample Selection and Data Sources

We select A-share listed companies on the Shanghai Stock Exchange and Shenzhen Stock Exchange from 2010 to 2019 as our research object. The metrics of internal control quality are taken from the DIB database; other data come from the CSMAR database. To ensure the representativeness of the data and the reliability of the research results, the original data are treated as follows, according to the salient research conventions:

The samples of ST and ST^*∗*^ enterprises with abnormal operation and delisting risk are removed. The samples with no R&D expenditure and missing internal control data are removed. The samples of listed companies in the financial sector are removed.

Finally, 753 research samples are generated, and 7530 examples of observational data are obtained.

### 3.2. Variable Definitions

1. Explained variable: R&D investment intensity (R&D). Existing studies mainly measure R&D investment intensity via income statements and balance sheets. Concerning income statement data, it is expressed as the value of the total R&D expenditure divided by the total sales revenue. Based on the declaration of high-tech enterprises, whether the proportion of R&D expenses to sales revenue meets the requirements is used for declaration. In the declaration of high-tech enterprises, the higher the revenues are, the higher the R&D expenses are. This can be understood as follows: the greater the investment in R&D is, the higher the gold content of products is. Therefore, the value of the total R&D expenditure divided by total sales revenue can be used as a measure of R&D investment intensity. Regarding balance sheets, however, R&D intensity is measured by dividing total R&D expenditures by total assets at the beginning of a period, which has a slight advantage over the former. On the one hand, the sales revenue of enterprises of different natures and industries will be very different. In some enterprises with little sales revenue, even if the R&D expenditure is similar to that of other enterprises, the intensity of R&D investment will be magnified because of the denominator, which contrasts with the actual situation. To reduce the degree of “incomparability,” at this point sales revenue should not be considered a measure of R&D investment intensity. On the other hand, the capital of R&D expenditure comes from the total assets of an enterprise, and the expenses that meet the conditions in the development stage can be capitalized and accounted for in intangible assets. Selecting the total assets at the beginning of a period can prevent double calculation because of the intangible assets that are included in the expenses caused by capitalization. Because our research object extends beyond high-tech enterprises and, to widely measure the R&D investment intensity of various enterprises, we decided to follow the research method of Yuan and Wang [30], we adopt the value of the total R&D expenditure of listed companies in each year divided by the total assets at the beginning of the period to express R&D investment intensity (R&D). The higher the value is, the greater the R&D investment intensity is, and the greater preference companies will have for R&D activities.
2. Explanatory variable: internal control quality (ICQ). There are approximately two methods to measure the quality of internal control: first, to use the internal control information disclosed by a company to design measurement methods and then examine them; second, to assess all kinds of relevant information and data of a company via a series of fair judgment standards with the aid of an independent third party to obtain a representative, comprehensive index of internal control and then treat the index mathematically according to a variety of research needs. Given our objective and practical focus, the second method was adopted in this paper. The comprehensive index of internal control released by Shenzhen DIB Enterprise Risk Management Technology Co., LTD. (hereafter DIB) was used as the measurement index of the quality of internal control, and the natural logarithm conversion method was utilized to reduce the difficulty of data processing. The comprehensive index of internal control issued by DIB is relatively authoritative in China. It covers the main contents related to the internal control of a target company that can objectively and fairly reflect the effectiveness of its internal control operation. The value of the index under natural logarithm transformation is larger, indicating that the quality of internal control is higher.
3. Intermediate variable: cash-holding level (Cash). We used the value of the sum of monetary capital and trading financial assets of listed companies divided by total assets to represent their cash-holding level. The higher the value is, the higher the corporate cash holdings are.
4. Control variables. Based on existing studies, we selected the following indicators that have a great impact on R&D investment intensity as our control variables: First is enterprise size (Size), which reflects the overall status of enterprise assets and is the strength guarantee of R&D and innovation activities. Our use of it as a control variable is mainly based on the influence of enterprise size on R&D investment intensity. Second is enterprise growth (Growth); the growth rate of operating income reflects the growth of enterprises to a certain extent. To achieve sustainable growth, innovative enterprises often carry out R&D activities; thus, the influence of operating income growth rate on R&D investment intensity needs to be controlled for. Third is corporate profitability (ROA); using the rate of return on assets to measure enterprise profitability can eliminate the influence of different company sizes on the research. The higher the rate of return on assets is, the higher the return on investment is, and the more positive the signal of R&D investment to decision-makers is, making them more optimistic about R&D investment; hence, the rate of return on assets was added to the control variable. Fourth is government R&D subsidy (SUB); since the government's financial subsidy in R&D and innovation helps enterprises carry out R&D activities, it was added to the research model. Our specific variable divisions and definitions are shown in Table 1.

### 3.3. Model Building

**Figure 1:**
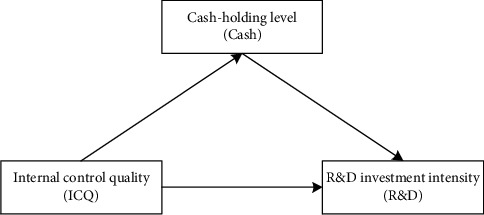
Mediation diagram.

In this paper, our main focus is on the influence of internal control quality on R&D investment intensity. Thus, we use cash-holding level as a mediating variable to further study the influence of internal control quality on R&D investment intensity through its influence on cash-holding level. Here, cash-holding level has a mediating effect. The relationship among the three is shown in Figure 1.

To analyze the impact of internal control quality on R&D investment intensity, we constructed the following regression model:

(1) $$\begin{array}{c} R\&D=\alpha +\beta \;\ln\;ICQ+\gamma \text{Controls}+\mu . \end{array}$$

In the above formula, *Controls* represents the set of control variables, including enterprise size (ln Size), enterprise growth (Growth), corporate profitability (ROA), and government R&D subsidy (ln SUB); *μ* represents the random error term. In Formula (1), the coefficient *β* of internal control quality (ln ICQ) is the focus of the study, which is used to measure the impact of internal control quality on R&D investment intensity. If H_1_ is true, *β* will be significantly positive in the following analysis; that is, improving internal control quality can promote the increase in R&D investment intensity.

According to the research of Sobel [31], the mediating effect of cash-holding level should meet the following conditions: The internal control quality can significantly affect the R&D investment intensity; the internal control quality can significantly affect the cash-holding level; the cash-holding level can significantly influence the R&D investment intensity. Through our evaluation and analysis of Equation (1), we are able to meet condition. In addition, the following mediation effect model should be constructed to achieve the remaining conditions:

(2) $$\begin{array}{c} \text{Cash}=\alpha_{1}+\beta_{1}\ln\;ICQ+\gamma_{1}\text{Controls}+\mu_{1}, \end{array}$$

(3) $$\begin{array}{c} R\&D=\alpha_{2}+\beta_{2}\ln\;ICQ+\lambda \text{Cash}+\gamma_{2}\text{Controls}+\mu_{2}. \end{array}$$

Formula (2) is used to investigate the relationship between internal control quality (ICQ) and cash-holding level (Cash), and Formula (3) introduces cash-holding level (Cash) based on Formula (1) to test the mediating effect of cash-holding level.

Accordingly, we test the regression coefficient step by step [32]. First, we test whether the internal control quality in Formula (1) positively affects R&D investment intensity, that is, whether the coefficient *β* is greater than zero and the significance level is high. Second, we show the relationship between the internal control quality and the corporate cash-holding level to test whether the coefficient *β*_1_ is significant. Finally, we examine the influence of the corporate cash-holding level and internal control quality on the R&D investment intensity. If both *β*_2_ and *λ* are significant and *β*_2_ is reduced compared with *β*, this indicates that the corporate cash-holding level has a positive mediating effect on the internal control quality of R&D investment, which illustrates that internal control quality not only has a significant impact on R&D investment but also indirectly affects R&D investment intensity through cash-holding level.

## 4. Analysis of Results

### 4.1. Descriptive Statistical Data Analysis

As Table 2 shows, strikingly, the minimum value of enterprise R&D investment intensity (R&D) is 0.0002, which is nearly zero and suggests that almost no capital is spent on R&D investment involving tens of millions in assets. In contrast, 24% of the assets of the enterprises with the highest R&D investment intensity are devoted to R&D activities, indicating that the investment in R&D activities by Chinese enterprises is extreme. Meanwhile, the average value is less than half of the maximum value, and the median value is less than the average value, indicating that, in the selected sample of listed companies, only a small part of enterprises have high R&D investment intensity and that more than half of companies have R&D investment intensity of less than 2%. According to international evaluation standards, when R&D investment accounts for 1% of the total assets at the beginning of a period, enterprises will find it very difficult to survive in a fierce market environment due to their lack of core competitiveness. When the proportion reaches 2%, enterprises can maintain basic survival. When the proportion reaches 5%, enterprises can occupy a favorable position in their market. Of course, this is the international evaluation standard. Domestic R&D investment is lower than foreign investment, so the proportion can be appropriately reduced. However, it still has good reference significance. Hence, according to the data description, the R&D investment intensity of general enterprises is only at the basic survival level; a considerable number of enterprises may struggle to survive in fierce markets because they do not have advantages. Enterprises play an important role not only in driving national economic development but also in leading innovation. Therefore, enterprises need to increase R&D investment intensity, gain competitive advantages, and improve competitiveness to survive stably in their market and contribute to national scientific and technological innovation.

Given the data on internal control quality (ln ICQ), we find that although the quality of internal control varies among different enterprises, the median value is greater than the mean value, indicating that the internal control quality of most enterprises is higher than the average level. Due to the 2011 regulatory measures for the internal control of listed companies, listed companies are required to gradually disclose internal control information under the strict supervision and inspection of the CSRC and other institutions. There is no way to conceal problems of internal control. If a listed company does not take measures to resolve its internal control defects, it may present unresolved problem information to the public. Due to external pressure, listed companies have to address their shortcomings in internal control by making adjustments and repairing loopholes in a timely manner. In the long run, the quality of internal control of listed companies will thus be gradually improved.

The maximum value of cash-holding level (Cash) is 0.831, and the minimum value is 0.002. There are significant differences among different enterprises. The median value of 0.139 is lower than the mean value of 0.167, indicating that the cash-holding level of most enterprises is lower than the average level. Amid imperfect capital market development, external financing costs are high, and R&D innovation projects need a large amount of funds to be operated smoothly. Therefore, a reasonable decision can be made to increase cash-holding level to augment R&D investment intensity.

The mean value of enterprise size (ln Size) is 22.826, the maximum value is 28.636, and the minimum value is 18.833. Generally, amid strict listing conditions, an enterprise asset scale may reach a certain level, so there is not much difference in the sizes of listed companies. The average growth rate of the companies (Growth) is 49.8%, and the maximum value is as high as 1878.372. Although the growth degree of listed companies is very different, the overall growth level is good. The average rate of return on assets (ROA) is 3.9%, indicating that profitability is not high. The average value of a government subsidy (ln SUB) is 16.243, the maximum value is 23.145, and the minimum value is 5.218. The minimum value is less than half of the average value, indicating that the amount of a government subsidy obtained by listed companies varies greatly.

### 4.2. Correlation Analysis

Correlation analysis between variables is shown in Table 3. First, it can be seen from the results in the table that internal control quality (ICQ), cash-holding level (Cash), and R&D investment intensity (R&D) are positively correlated in pairs, and the correlation coefficient is significant at the 1% level, which can preliminarily confirm the correctness of the hypothesis and the reliability of the data. Second, R&D investment intensity (R&D) is correlated with each control variable at the 1% significance level. In particular, it is noted that three control variables are negatively correlated with R&D investment intensity (R&D): enterprise size (Size), enterprise growth (Growth), and enterprise profitability (ROA), indicating that large companies with strong profitability may have strong market competitiveness but weak innovation consciousness. The positive correlation between R&D investment intensity (R&D) and government R&D subsidy (SUB) indicates that government subsidy for R&D projects can stimulate enterprises' R&D investment activities. Third, the correlation coefficients among all variables are less than 0.5, proving that the selected control variables will not have a significant impact on the study of the main variables, which is reasonable. In general, the correlation coefficients of all variables are in an acceptable range, and there will not be serious multicollinearity, which will affect the feasibility of the study, in the subsequent multiple regression analysis.

### 4.3. Regression Analysis

The regression analysis results are shown in Table 4. The three models are the results of regression analysis on the basis of controlling for enterprise characteristics and related variables. Model 1 shows that the influence coefficient of ICQ on R&D is *β* = 0.0024 and that there is a positive correlation between ICQ and R&D at the significance level of 1%. This indicates that the higher the quality of internal control is, the better the enterprise management level is, thereby increasing R&D investment. Therefore, Hypothesis 1 is verified. The establishment of H_1_ also provides the basic conditions for our subsequent research.

Model 2 and Model 3 analyze whether cash-holding level has a mediating effect. In Model 2, the influence coefficient of ICQ on Cash is *β*_1_ = 0.0302, and it has a significant influence at the 1% level, indicating that internal control quality is positively correlated with corporate cash holdings. Thus, in a high-quality internal control environment, the cash holdings of an enterprise can reach a reasonably high level, verifying H_2_.

Model 3 studies the joint influence of internal control quality and cash-holding level on R&D investment intensity. Cash-holding level is embedded in Model 1 as a mediating variable; hence, we form the new model to test not only the influence of cash-holding level on R&D investment intensity but also whether there is a mediating effect. In the column of Model 3, the coefficient *β*_2_ of internal control quality (ICQ) is 0.0021, the coefficient *λ* of cash-holding level (Cash) is 0.0109, and the significance level of both coefficients is 1%, indicating that cash-holding level is positively correlated with R&D investment intensity. Meanwhile, the value of *β*_2_ is smaller than that of *β* in Model 1; that is, the coefficient value of internal control quality in the mediating effect model is smaller than that of internal control quality in Model 1 (without introducing the mediating variable), indicating that the effect of internal control quality on R&D investment intensity is dispersed due to the intervention of cash-holding level. Thus, internal control quality has an effect on both R&D investment intensity and cash-holding level, while R&D investment intensity is affected by both internal control quality and cash-holding level. Cash-holding level therefore has a mediating effect, which verifies H_3_.

### 4.4. Robustness Test

To determine whether our selected measurement indices and evaluation methods can maintain a consistent and stable interpretation of the research conclusions, the following robustness tests are conducted:

1. A robustness test is performed after changes are made to the metrics. Internal control quality is the most important variable in this study, and its measurement index is the natural logarithm of the internal control index of the DIB database. To make the study more rigorous, we replace the measurement of internal control quality and conduct a similar control test. Observing the annual reports of listed companies, we decide that if listed companies disclose internal control audit reports and these audit reports show no major defects, the value of the internal control quality is 1; otherwise, it is 0. The regression analysis results via this replacement are shown in Table 5. The correlation coefficients are still significant, which proves that the adopted internal control measures will not affect the stability of the regression results.
2. A robustness test is carried out after enterprise clustering. As an important manifestation of the relevant correlation, group structure cannot be ignored. To prove that the research results are not affected by the clustering effect, the sample enterprises are tested after clustering, as shown in Table 6. Each correlation coefficient is significantly positively correlated at the level of 1%, which is consistent with the above empirical results, proving that the conclusions of this study have good stability.

## 5. Conclusion and Implications

### 5.1. Main Conclusion

Based on the data of China's A-share listed companies from 2010 to 2019, in this paper, we empirically analyze the correlation between internal control quality and R&D investment intensity with cash-holding level as an intermediary factor. Our results show that internal control quality has a positive correlation with R&D investment intensity and that, due to the mediating effect of cash-holding level, internal control quality can indirectly promote the development of R&D activities by positively influencing cash-holding level. Given our findings on the role of cash-holding level as a mediator variable, this study enriches the relevant literature on the improvement effect of internal control quality and the influencing factors of R&D investment intensity. In addition to proving the positive correlation between internal control quality and R&D investment intensity, the influence mechanism between the two is clarified; that is, we demonstrate the transmission path of “internal control quality⟶cash-holding level⟶R&D investment intensity,” which has both theoretical significance and practical value.

Our research and analysis in this paper also reveal that the R&D investment level of most enterprises is lower than the average level. In a fierce market competition environment, the benefits for an enterprise only maintain its basic survival, and some enterprises even struggle to survive. Innovative R&D activities are characterized by a long cycle, high cost, high risk, and uncertain return, which often discourages many companies from investing in R&D. However, if enterprises want to become increasingly stronger in the new era, they must not abandon product or technology innovation. Although there are many factors that affect R&D investment intensity, such as operating performance, financing constraints, debt financing cost, company size, company growth, or social responsibility fulfillment degree, R&D investment intensity is mainly limited by cash holdings. However, the cash-holding level of enterprises will be affected by their internal control quality. Therefore, enterprise innovation should be supported by R&D investment, while R&D investment intensity can be increased by improving internal control quality and cash-holding level.

### 5.2. Implications

Based on the above conclusions, this paper provides the following insights:

1. To effectively guarantee high R&D investment intensity and drive enterprise innovation enthusiasm, the quality of enterprise internal control should be improved. On the one hand, from the perspective of management, the agency conflicts between management and shareholders can be alleviated amid effective internal control implementation. Both sides can reach consensus on R&D investment decisions and set a clear goal for R&D activities. In terms of making management decision-making behavior more transparent, management's self-interest will not be easily shaken. Moreover, an improvement in internal control quality can benefit management through objective and just incentive systems, ensuring that invested funds will be truly and effectively used in R&D [33]. On the other hand, regarding capital, the funding sources of R&D include current operating cash flows, government subsidies, and internal and external financing. As shown in the above theoretical analysis, enterprises are finding it increasingly difficult to obtain external financing, and government subsidies cannot benefit every enterprise; thus, the “burden” supporting R&D investment is largely internal funds, even cash with strong liquidity. If the funds used for R&D are not well controlled internally, the self-interested motives and investment distortion behavior of enterprise management will lead to an improper or unreasonable allocation of cash resources for R&D, which may reduce the intensity of R&D investment and weaken the power of R&D. As a key link in internal control activities, the high-quality management of cash flow can therefore effectively solve the problem of fund allocation. Nevertheless, limiting this to cash flow management is, of course, not enough. Each unit of internal control is interlinked, and the management of other units will also have an impact on cash management. Enterprises should thus pay attention to improving their overall quality of internal control, coordinating the management of all unit activities, clarifying the relevant factors of R&D investment, and making reasonable arrangements for R&D activities to not only guarantee a certain intensity of R&D investment but also ensure the sustainability of R&D investment activities.
2. Enterprises should establish mechanisms for R&D and innovation activities and strive to achieve the automatic vitality of R&D. This research shows that there is a positive correlation between cash-holding level and R&D investment intensity and that a high cash-holding level can have a positive effect on the promoting effect of internal control on R&D investment. Companies can combine internal control quality, R&D investment intensity, and cash-holding level to build front and back linkage mechanisms by applying these findings. The former mechanism is “internal control quality⟶cash-holding level,” whose goal is to design rules and guidelines that facilitate improving the quality of internal control to improve the cash-holding level for all members of an enterprise to abide by. This mechanism should help companies strengthen their cash flow management in their internal control by, for example, setting their optimal cash-holding level reasonably and maximizing their use of cash holdings to achieve the cash-holding level required by subsequent research and development. This is followed by the mechanism of “cash-holding level⟶R&D investment intensity,” whose ultimate goal is improving R&D investment intensity. This mechanism provides sufficient power to R&D activities by restraining the selfishness of senior management and other negative behaviors that are detrimental to R&D activities, for example, planning the correct direction for the use of cash flow by controlling both mechanisms simultaneously and strengthening the connectivity between the front and back. Thus, R&D investment decisions can follow a clear forward direction, be defended on this path, and reap considerable benefits through the effective operation of internal control and a high cash-holding level, thereby further stimulating the internal control of R&D investment. In addition, internal control can promote improving cash-holding level, fostering the whole mechanism at every step, and presenting a virtuous cycle, which not only achieves the purpose of enhancing the intensity of R&D investment but also ensures the continuity of R&D investment activities.
3. According to the different stages of enterprise development, an appropriate internal control operation scheme needs to be designed, and R&D investment intensity needs to be dynamically adjusted. In the initial stage, enterprises are generally unable to carry out large R&D investment but have strong R&D willingness. In the growth stage, with the support of capital, research and development can be realized, and an enterprise may rely on these research and development results to obtain rapid growth. Mature companies may fall into an R&D slump, when their R&D activities may be affected by a variety of factors and become stagnant, despite their deep pockets. Additionally, enterprises should be alert to the arrival of a recession period. Innovation is an important means to prevent a recession and extend enterprise life, and the source and power of enterprise innovation is R&D investment. During continuous development, the resource scale and management methods of enterprises are constantly changing, and the status of their R&D investment is no exception. In our data analysis, we have found that the impact of internal control quality on R&D investment intensity may change with the development stage of an enterprise. Hence, the construction and implementation of internal control does not occur on a single occasion. Enterprises should thus focus on the construction of management mechanisms related to R&D projects in their implementation of research and development projects. Moreover, they should continue to pay attention to the changes of elements in their mechanism and investigate whether there are contradictions to dynamically improve their internal control, to more effectively promote their investment in innovative research and development.

### 5.3. Research Deficiencies and Prospects

Although this paper studies the relationship among internal control quality, cash-holding level, and R&D investment intensity, it has the following shortcomings: The samples that we selected in this paper are listed companies on the main board rather than on the GEM and SME boards, because China forces GEM and SME board listed companies to implement internal control at a later time. Their internal control quality characteristics are thus different from those of listed companies on the main board. To avoid interfering factors, we have excluded them. In the future, listed companies on the GEM and SME boards can be selected as samples to further study the influence of internal control quality on the R&D investment intensity of enterprises. In addition, cash-holding level is analyzed as an intermediary variable in this paper, as cash management is one of many elements of internal control. The enhancement of the quality of internal control requires a balanced improvement in all elements. Accordingly, how other elements of internal control affect the R&D investment intensity of enterprises also needs further research.

## Figures and Tables

**Table 1:** Variable definitions.

Variable type	Variable name	Variable symbol	Variable declaration
Explained variable	R&D investment intensity	*R&D*	R&D expenditure/total assets at the beginning
Explanatory variable	Internal control quality	*ICQ*	Natural logarithm of the DIB database internal control index
Intervening variable	Cash-holding level	*Cash*	(Monetary capital + tradable financial assets)/total assets
Control variable	Enterprise size	*Size*	Natural logarithm of the total assets
	Enterprise growth	*Growth*	Growth rate of operating income
	Corporate profitability	*ROA*	Net profit/total assets
	Government R&D subsidy	*SUB*	Natural logarithm of the government subsidy

**Table 2:** Descriptive statistics.

Variable	Minimum	Maximum	Average	Standard deviation	Median
R&D	0.0002	0.240	0.026	0.007	0.014
ln ICQ	2.194	6.903	6.510	0.146	6.524
Cash	0.002	0.831	0.167	0.115	0.139
ln Size	18.833	28.636	22.826	1.434	22.684
Growth	−0.947	1878.372	0.498	21.766	0.093
ROA	−0.612	2.637	0.039	0.058	0.032
ln SUB	5.218	23.145	16.243	2.205	16.419

**Table 3:** Correlation analysis.

Variable	R&D	ICQ	Cash	Size	Growth	ROA	SUB
R&D	1.0000						
ICQ	0.0142^*∗∗∗*^	1.0000					
Cash	0.0143^*∗∗∗*^	0.1416^*∗∗∗*^	1.0000				
Size	−0.0292^*∗∗∗*^	0.2842^*∗∗∗*^	−0.1829^*∗∗∗*^	1.0000			
Growth	−0.0034^*∗∗∗*^	−0.0350^*∗∗∗*^	−0.0106^*∗∗∗*^	0.0004^*∗∗∗*^	1.0000		
ROA	−0.0027^*∗∗∗*^	0.0987^*∗∗∗*^	0.1713^*∗∗∗*^	−0.013^*∗∗*^	−0.0015^*∗*^	1.0000	
SUB	0.0146^*∗∗∗*^	0.0023^*∗∗*^	0.0037^*∗∗*^	0.0260^*∗∗*^	0.0465^*∗∗*^	0.0632^*∗∗*^	1.0000

*Note.* ^*∗*^, ^*∗∗*^, and ^*∗∗∗*^ represent the significance levels of the coefficients at 10%, 5%, and 1%, respectively. The smaller the percentage value is, the more significant the relationship is.

**Table 4:** Regression analysis.

Variable	Model 1 R&D	Model 2 Cash	Model 3 R&D
ICQ	0.0024^*∗∗∗*^ (4.08)	0.0302^*∗∗∗*^ (3.28)	0.0021^*∗∗∗*^ (3.48)
Cash			0.0109^*∗∗∗*^ (4.29)
Size	−0.0007^*∗∗∗*^ (−4.18)	−0.0153^*∗∗∗*^ (−16.47)	−0.0007^*∗∗∗*^ (−4.04)
Growth	−0.0002^*∗∗∗*^ (−5.98)	−0.0001^*∗∗∗*^ (−0.81)	−0.0001^*∗∗∗*^ (−5.64)
ROA	−0.0029^*∗∗∗*^ (−2.12)	0.3249^*∗∗∗*^ (14.71)	−0.0026^*∗∗*^ (−2.09)
SUB	0.0134^*∗∗∗*^ (3.47)	0.0013^*∗∗*^ (5.88)	0.0128^*∗∗∗*^ (3.54)
Year	Control	Control	Control
Constant	0.1026^*∗∗∗*^ (7.13)	0.3079^*∗∗∗*^ (5.35)	0.1023^*∗∗∗*^ (10.23)
Observations	7530	7530	7530
*R* ^2^	0.0193	0.0635	0.0215

*Note.* ^*∗*^, ^*∗∗*^, and ^*∗∗∗*^ indicate that the coefficient significance levels are 10%, 5%, and 1%, respectively. The smaller the percentage value is, the more significant the relationship is. The data in parentheses are *T* values.

**Table 5:** Robustness test by changing the internal control measure index.

Variable	Model 1 R&D	Model 2 Cash	Model 3 R&D
ICQ	0.00098^*∗∗∗*^ (3.17)	0.0286^*∗∗∗*^ (3.00)	0.00096^*∗∗∗*^ (3.02)
Cash			0.0087^*∗∗∗*^ (3.76)
Constant	0.103^*∗∗∗*^ (9.04)	0.31^*∗∗∗*^ (6.83)	0.104^*∗∗∗*^ (10.98)
Observations	7530	7530	7530
*R* ^2^	0.0183	0.0622	0.0198

*Note.* ^*∗*^, ^*∗∗*^, and ^*∗∗∗*^ indicate that the coefficient significance levels are 10%, 5%, and 1%, respectively. The smaller the percentage value is, the more significant the relationship is. The data in parentheses are *T* values.

**Table 6:** Robustness test by enterprise cluster.

Variable	Model 1 R&D	Model 2 Cash	Model 3 R&D
ICQ	0.0024^*∗∗∗*^ (3.97)	0.0302^*∗∗∗*^ (2.21)	0.0021^*∗∗∗*^ (3.29)
Cash			0.0098^*∗∗∗*^ (3.99)
Constant	0.0034^*∗∗∗*^ (8.04)	0.0028^*∗∗∗*^ (3.83)	0.1056^*∗∗∗*^ (8.98)
Observations	7530	7530	7530
*R* ^2^	0.0183	0.0622	0.0198

*Note.* ^*∗*^, ^*∗∗*^, and ^*∗∗∗*^ indicate that the coefficient significance levels are 10%, 5%, and 1%, respectively. The smaller the percentage value is, the more significant the relationship is. The data in parentheses are *T* values.

## Data Availability

DIB database is available at https://www.dibdata.cn/. CSMAR database is available at https://www.gtarsc.com/.
